# Both Freshly Prepared and Frozen-Stored Amniotic Membrane Cells Express the Complement Inhibitor CD59

**DOI:** 10.1100/2012/815615

**Published:** 2012-05-22

**Authors:** Ágnes Füst, Éva Pállinger, Adrienn Stündl, Eszter Kovács, László Imre, Sára Tóth, János Németh

**Affiliations:** ^1^Department of Ophthalmology, Semmelweis University, Tömő u. 25-29, Budapest H-1083, Hungary; ^2^Research Group for Inflammation Biology and Immunogenomics of Hungarian Academy of Sciences and Semmelweis University, Nagyvárad tér 4, Budapest H-1089, Hungary; ^3^1st Department of Obstetrics and Gynecology, Semmelweis University, Baross u. 27, Budapest H-1088, Hungary

## Abstract

Amniotic membrane proved to be very effective tool in the treatment of a number of ocular surface diseases. The amniotic membrane, however, has to be stored before its transplantation onto the ocular surface followed by mandatory serologic control in order to exclude the transmission of certain viruses. Therefore it is most important to study if cryopreservation of the membrane affects cell surface expression of the molecules. We measured cell surface expression of CD59, a membrane-bound complement inhibitor on the cells of freshly prepared and cryopreserved amniotic membrane. Cells of amniotic membrane were separated mechanically. Epithelial and mesenchymal cells were identified by the intracellular expression of nanog and the cell surface ICAM1 positivity, respectively. Multicolor flow cytometric immunophenotyping was used for determination of the CD59 expression. CellQuest-Pro software program (Becton Dickinson) was used both for measurements and analysis. CD59-positive cells could be detected in all investigated samples and in all investigated cell types, although the expression level of CD59 differed. CD59 was expressed both on freshly prepared and frozen-stored samples. Higher level of CD59 was detected on ICAM1+ mesenchymal cells than on nanog+ epithelial cells. Our findings indicate that amniotic membranes maintain their complement inhibiting capacity after cryopreservation.

## 1. Introduction

Amniotic membrane covers the innermost layer of the fetal extraembryonic membrane. The amniotic membrane consists of a single layer of columnar epithelial cells, a thick basement membrane, and a thin layer of avascular stroma. In the last 15 years the amniotic membrane has been used widely in the ophthalmology. It proved to be very effective tool in the treatment of a number of ocular surface diseases, such as nonhealing epithelial defect and sterile ulcer of the cornea, alkali burn of the eye, and surgery of cicatrizing conjunctivitis [1]. One of the most valuable properties which makes the amniotic membrane suitable for this is its intrinsic anti-inflammatory action [1]. The mechanism of this anti-inflammatory, immunosuppressive effect is not completely understood. A complement inhibitor capacity could contribute to the anti-inflammatory effect of the transplanted amniotic membrane.

Complement activation products C3a, C4a, and C5a were detected in the amniotic fluid samples of normal pregnants, suggesting that these anaphylatoxins are physiologic constituents of the amniotic fluid [2–5]. The increased activation of the complement system as a consequence of microbial invasion may lead to preterm labor [4] Therefore the protection of extraembryonic membranes from the action of the activated complement components of the amniotic fluid is essential [6].

CD59 is a surface-bound complement inhibitor protein; its main role is to prevent the “self” cells from lysis by the activated complement system. It acts by preventing the formation of the membrane attack complex at the terminal step of the complement activation cascade. Due to its crucial role in preventing damage to “self” cells, it is widely expressed, found in a lot of tissues in the body, and is present on all circulating cells [7]. The lack of CD59 has a fundamental pathogenetic role in a potentially life-threatening disease of the blood, the paroxysmal nocturnal hemoglobinuria, which can be treated successfully with the complement-inhibitor eculizimab [8]. The expression of CD59 has previously been detected both on epithelial [6, 9] and on mesenchymal [10, 11] cells of the fresh amniotic membrane, too. 

However, because of the mandatory serologic control to exclude the transmission of certain viruses, the amniotic membrane has to be stored for at least 6 months before its transplantation onto the ocular surface. Therefore our aim was to study if cryopreservation influences the expression of CD59 on the cells of the amniotic membrane.

## 2. Materials and Methods

### 2.1. Amniotic Membrane Samples

The preparation of the amniotic membrane was performed under sterile conditions according to the method of Kim and Tseng [12]. The amniotic membrane was mechanically separated from the placenta taken from normal full-term uncomplicated elective cesarean section. The separated amnion tissue was thoroughly washed in phosphate-buffered saline that contains antibiotics to cover Gram-negative and Gram-positive bacteria and antifungal drugs. The pieces of membrane were spread epithelial side up on nitrocellulose paper and fixed with two 7/0 vicryl sutures. The pieces for storage were placed in small bottles in fluid containing 50% glycerol and 50% Dulbecco's modified Eagle Medium (DMEM, Gibco). The tissue was stored frozen at −80°C for 1 to 14 months. 

Investigated amnion membrane samples derived from 9 different placentas. For the investigation of the effects of cryopreservation we detected the CD59 expression of amniotic cells in freshly prepared membranes and from amniotic membrane samples stored for 1 to 14 months. The number of samples taken from a certain placenta at a certain preservation time was 1 to 5 (Table 1).

A piece amniotic membrane of a placenta was used for observing the effect of 15 minutes freezing at −80°C, the cells isolated from half of the native sample and from the frozen second half of the same sample were investigated simultaneously.

### 2.2. Separation of the Cells from the Amniotic Membrane Pieces

The separation of the amniotic membrane cells was performed mechanically with the medimachine device (Becton Dickinson). The advantage of this mechanical separation over the enzymatic separation is its tolerance of the cells, that is, the proportion of cells remaining intact is high. For the cell separation a 35 *μ*m pore size milling head was used. The machine worked for 45 seconds. After that the cell suspension was removed from the milling head with syringe and filtered through 35 *μ*m pore size Filcon filter.

### 2.3. Multicolor Staining of Cells for Flow Cytometry

CD59 expression of amnion membrane cells that was determined by fluorescein isothianate (FITC) conjugated monoclonal anti-human CD59 antibody. Intracellular staining of nanog (a transcription factor critically involved with self-renewal of undifferentiated embryonic stem cells) was used for the identification of epithelial cells, while cell surface ICAM1 (CD54) positivity was used for the characterization of mesenchymal cells [13].

For the detection of CD59 expression of mesenchymal cells, 10^5^ cells were incubated with FITC-conjugated monoclonal anti-human CD59 antibody and phycoerythrin (Pe-) conjugated monoclonal antihuman ICAM1 (CD54) antibody at room temperature for 20 minutes. Both antibodies were manufactured by BD Biosciences (San Jose, CA, USA). After incubation the unbound antibodies were removed by washing with PBS and the cells were fixed by 2% paraformaldehyde (Sigma-Aldrich, St. Louis, MO, USA) solution. For the determination of CD59 expression of epithelial cells, the CD59 stained cells were fixed by 4% paraformaldehyde (Sigma-Aldrich, St. Louis, MO, USA), permeabilized by 0.1% saponin solution (Sigma) and incubated with Pe-conjugated anti-human nanog monoclonal antibody. Before the measurements cells were washed with 0.1% saponin solution for removing the unbound antibodies and fixed by 2% paraformaldehyde (Sigma-Aldrich, St. Louis, MO, USA) solution. Optimal amounts of antibodies were determined earlier.

### 2.4. Measurements

**Figure 1:**
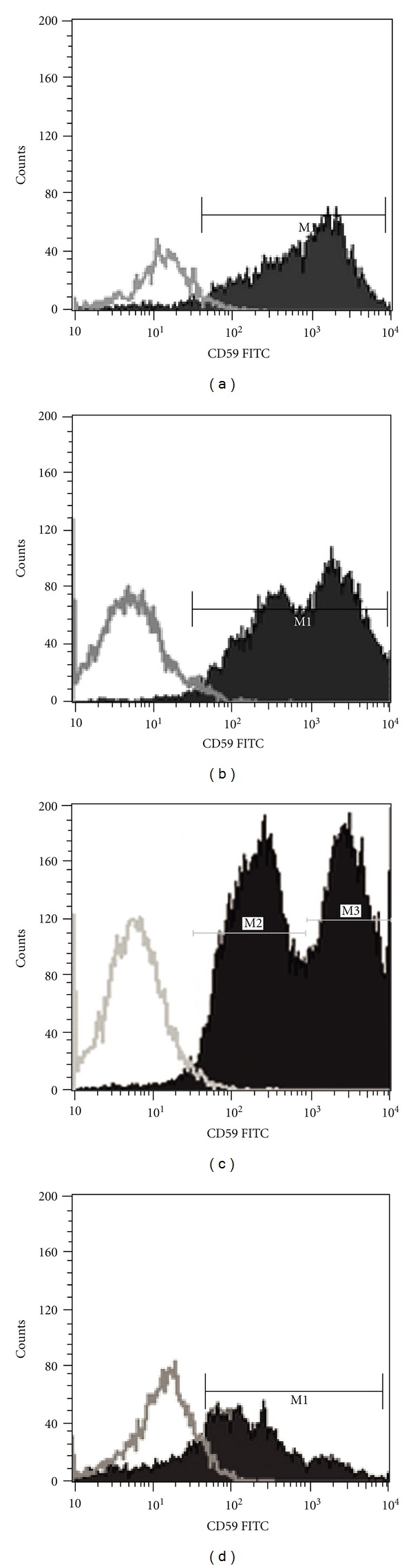
CD59 expression of amniotic cells. Count of the CD59+ cells versus the intensity of cell staining is presented. (a): fresh, (b): 1 month, (c): 4 months, (d): 14 months freezing time. The intervals signed by M1 marker mark out the CD59-positive cells. M2 and M3 show the subgroups of less intensively and more intensively stained cells, respectively.

Measurements were carried out using a FACS Calibur flow cytometer (Becton Dickinson) on the day of the staining, collecting 5 × 10^4^ cells/tube. CellQuest-Pro software program (Becton Dickinson) was used for analysis. Detected curves (histograms) were separated based on their fluorescence intensity; control peak derived from “isotype” control antibodies, while positive histogram peaks derived from specific antibodies. Quantitative percentage of cells with the investigated properties was calculated by the software after separated the histograms with markers (see Figure 1, M1 marker).

### 2.5. Statistical Analysis

Kruskal-Wallis nonparametric analysis of variance was performed to compare the percentage of the CD59+ cells of the freshly prepared and cryopreserved samples.

## 3. Results

### 3.1. Effect of Cryopreservation on the CD59 Expression of the Amniotic Membranes

CD59-positive cells could be detected in all investigated amniotic membrane samples. When we examined the same fresh sample before and after 15 minutes freezing, differences could not been detected in the fluorescence intensities (data not shown). The percentage of the CD59+ cells varied among both the freshly prepared and cryopreserved (1 to 14 months) samples (46–94% and 39–98%, resp.). There was, however, no statistically significant difference in the proportion of the CD59+ cells between the fresh and preserved samples (Table 2).

The percentage of CD59+ cells was variable even among different pieces of the same placenta at the same preservation time. The variation coefficient of the measurement in fresh placentas was 22.9%.

### 3.2. Expression of CD59 on Different Cell Types of the Amniotic Membranes

**Figure 2:**
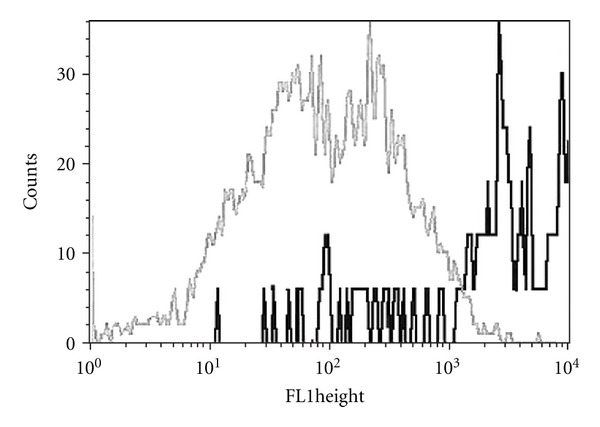
CD59 expression of epithelial and mesenchymal cells. Count of nanog+ epithelial cells and ICAM+ mesenchymal cells versus intensity of CD59 positivity is shown. Lower level of CD59 could be detected on the nanog+ epithelial cells than on ICAM1+ mesenchymal cells. (grey color: nanog+ epithelial cells, dark color: ICAM+ mesenchymal cells).

 The detected CD59 histograms frequently show a bimodal (2 peak) distribution (Figure 1). Amniotic membranes contain different cell types, most of them are epithelial cells and mesenchymal cells. We supposed that the bimodal histograms were caused by the different expression levels of CD59 on different amniotic cell types. Using multicolor flow cytometric measurements we compared the cell surface expression of CD59 of epithelial and mesenchymal cells. CD59 were found to be expressed in both types of cells. Higher level of CD59, however, could be detected on ICAM1+ (CD54) mesenchymal cells than on the nanog+ epithelial cells (Figure 2).

## 4. Discussion

In the present study we examined whether the amniotic membrane retains its complement inhibitory potential, after storage frozen for 6 months in the form it is usually sutured on the diseased ocular surface. We demonstrated the presence of complement regulator CD59 on the surface of freshly prepared and cryopreserved amniotic cells as well. The cells of the amniotic membrane do not survive the freezing at −80°C, no living cells were detected with vital staining and ultrastructural examination, and cells removed enzymatically from cryopreserved membranes did not grow in culture [14, 15]. However, our results show that the CD59 molecules produced originally are still present on the surface of these cells after freezing, so they have the capability to express its complement regulatory effect.

Complement system is a powerful inflammatory agent, which certainly has role in the defense of the ocular surface. We understand complement activation under certain physiologic circumstances, like closed eye [16, 17] and some nonphysiologic or pathologic conditions, such as contact lens wear [18], after keratoplasty [19] and in conjunctivitis [20]. The elements of the complement system were detected in the normal human cornea as well [21]. However, in state of increased complement activation, ocular surface needs protection. In deed, large amount of complement inhibitor factors (CD59, DAF) was shown out on the surface of the cornea and conjunctiva [22]. This defense mechanism may not be enough for preserving the ocular surface integrity in case of some conditions such as nonhealing epithelial defect and ulcer of the cornea. The presence of CD59 in the transplanted cryopreserved amniotic membrane could supplement the autologous protection of the ocular surface. This could contribute to the explanation of the beneficial effect of the amniotic membrane in the ocular surface diseases. As the CD59 is bounded both to the epithelial and stromal cells of the amniotic membrane, this may contribute to the fact that the anti-inflammatory effect of the amniotic membrane is independent from that it is positioned onto the ocular surface with epithelial side up or down.

Another novel finding of our study is the difference between the extent of CD59 expression between two types of cells of the amniotic membrane, epithelial and mesenchymal cells. In earlier studies CD59 expression was demonstrated both on epithelial and mesenchymal cells, but they were not examined in the same experiment [6, 9–11]. Using multicolor staining method in the present study we could compare the cell surface expression of CD59 on epithelial cells and mesenchymal cells, in the same sample. We could demonstrate that the mesenchymal cells express higher amounts of CD59 on their surface than the epithelial cells.

We can conclude that the amniotic membrane derived epithelial and mesenchymal cells express in different extent the complement inhibitory protein CD59 molecules on their surface. CD59 expression could be detected both on freshly prepared samples and also after cryopreservation, indicating that amniotic membranes maintain their complement inhibiting ability during cryopreservation.

## Figures and Tables

**Table 1:** Number of amniotic membrane pieces taken from the certain placentas and worked up after different freezing times. The A–G marks show the 7 different placentas used for the experiment. The numbers show the pieces taken from the certain placenta and processed freshly or after the signed time long freezing.

Placenta	fresh	2–4 months	5–8 months	13-14 months
A				2
B			2	
C		2	1	
D	5			
E	1			4
F			4	
G		5		

**Table 2:** Rate of CD59 positive cells in amniotic membrane pieces processed after different freezing times. The rates are given in percent, as mean ± standard deviation. Markedly no relation can be discovered in the rate of CD59-positive cells and the freezing time.

Placenta	fresh	2–4 months	5–8 months	13-14 months
A				62, 0 ± 18, 7
B			88, 0 ± 11, 2	
C		96, 5 ± 0, 5	51	
D	63, 0 ± 14, 5			
E	94,0			61, 7 ± 15, 3
F			56, 1 ± 6, 8	
G		63, 6 ± 18, 5		
